# Cytokinin Detection during the *Dictyostelium discoideum* Life Cycle: Profiles Are Dynamic and Affect Cell Growth and Spore Germination

**DOI:** 10.3390/biom9110702

**Published:** 2019-11-05

**Authors:** Megan M. Aoki, Anna B. Kisiala, Shaojun Li, Naomi L. Stock, Craig R. Brunetti, Robert J. Huber, R. J. Neil Emery

**Affiliations:** 1 Department of Biology, Trent University, Peterborough, ON K9L 0G2 Canada; annakisiala@trentu.ca (A.B.K.); craigbrunetti@trentu.ca (C.R.B.); roberthuber@trentu.ca (R.J.H.); nemery@trentu.ca (R.J.N.E.); 2 Noblegen, Peterborough, ON K9L 0G2, Canada; shaojun.li@noblegen.com; 3 Water Quality Centre, Trent University, Peterborough, ON K9L 0G2, Canada; naomistock@trentu.ca

**Keywords:** ultra high-performance liquid chromatography-positive electrospray ionization-high resolution tandem mass spectrometry (UHPLC-(ESI+)-HRMS/MS), cytokinin, cytokinin biosynthesis, *Dictyostelium discoideum*, social amoeba, discadenine, germination

## Abstract

Cytokinins (CKs) are a family of evolutionarily conserved growth regulating hormones. While CKs are well-characterized in plant systems, these *N*^6^-substituted adenine derivatives are found in a variety of organisms beyond plants, including bacteria, fungi, mammals, and the social amoeba, *Dictyostelium discoideum*. Within *Dictyostelium*, CKs have only been studied in the late developmental stages of the life cycle, where they promote spore encapsulation and dormancy. In this study, we used ultra high-performance liquid chromatography-positive electrospray ionization-high resolution tandem mass spectrometry (UHPLC-(ESI+)-HRMS/MS) to profile CKs during the *Dictyostelium* life cycle: growth, aggregation, mound, slug, fruiting body, and germination. Comprehensive profiling revealed that *Dictyostelium* produces 6 CK forms (*cis*-Zeatin (*c*Z), discadenine (DA), *N*^6^-isopentenyladenine (iP), *N*^6^-isopentenyladenine-9-riboside (iPR), *N*^6^-isopentenyladenine-9-riboside-5′ phosphate (iPRP), and 2-methylthio-*N*^6^-isopentenyladenine (2MeSiP)) in varying abundance across the sampled life cycle stages, thus laying the foundation for the CK biosynthesis pathway to be defined in this organism. Interestingly, iP-type CKs were the most dominant CK analytes detected during growth and aggregation. Exogenous treatment of AX3 cells with various CK types revealed that iP was the only CK to promote the proliferation of cells in culture. In support of previous studies, metabolomics data revealed that DA is one of the most significantly upregulated small molecules during *Dictyostelium* development, and our data indicates that total CK levels are highest during germination. While much remains to be explored in *Dictyostelium*, this research offers new insight into the nature of CK biosynthesis, secretion, and function during *Dictyostelium* growth, development, and spore germination.

## 1. Introduction 

*Dictyostelium discoideum* is one of the most well-known representatives of the *Amoebozoa* phylum [1]. Owing to its unique life cycle, this social amoeba can exist both as a single-cellular and multicellular organism. In its natural environment, *Dictyostelium* amoebae feed upon bacteria and decaying leaf litter in the forest soil. However, once food resources are depleted, the developmental programme is initiated through dramatic transcriptional changes, thus enabling *Dictyostelium* to adapt to a period of starvation and subsequent transition to multicellularity [2]. cAMP is secreted as a chemical messenger in a pulsatile manner from the population of starved amoebae and acts as a chemoattractant causing the cells to migrate towards a central location [3,4]. Thousands of cells form aggregates, referred to as mounds. The cells in the mound continue to secrete cAMP at the apex, which sends a column of cells into the air that eventually topples over forming the pseudoplasmodium or slug [5]. Light and temperature guide the migration of the slug towards the top layer of the forest soil, where the fruiting body forms. This final structure consists of a slender tube-like stalk that suspends the spores above the surface for future germination and largely depends on cytokinins (CKs), which are signalling molecules that initiate spore formation and maintain spore dormancy [6,7,8].

CKs are a group of evolutionarily conserved *N*^6^-substituted adenine derivatives, most commonly known for their widespread signalling effects influencing nearly all aspects of plant growth and development [9]. Within *Dictyostelium*, 2 CK types have been identified to date, *N*^6^-isopentenyladenine (iP) and discadenine (DA) [10,11]. In plants and other CK-producing organisms (e.g., bacteria, fungi, and insects, etc.), iP is the precursor CK from which other CKs are derived [12,13]. iP is also the precursor molecule to DA, in which direct transfer of the 3-amino-3-carboxy-propyl moiety of *S*-adenosylmethionine (SAM) is added to the ***N***3 position of the adenine molecule [14]. DA is unique to social amoebae and has only been detected among Dictyostelids that secrete cAMP as their chemoattractant for aggregation [15,16,17]. DA was first discovered for its role as a potent spore germination inhibitor [7,8,16] and more recently for its role in inducing sporulation of *Dictyostelium* cells, along with iP and other exogenously applied CKs [6]. These developmental roles of CK are the only documented roles of CK in *Dictyostelium* to date.

Work by Anjard and Loomis [6] identified the primary CK biosynthesis gene in *Dictyostelium*, *iptA*, which is responsible for 90% of the CKs produced within the fruiting body. In CK-producing organisms, isopentenyltransferases (IPTs) are the key enzymes responsible for the formation of isoprenoid-type CKs, such as iP, dihydrozeatin (DZ), *trans-*zeatin (*t*Z), and *cis-*zeatin (*c*Z). There are 2 different forms of IPT: adenylate-IPTs and tRNA-IPTs [18]. Adenylate-IPTs produce free CKs through de novo biosynthesis pathway, whereas tRNA-IPTs produce CKs bound to tRNA. Upon tRNA-degradation, the bound CKs are liberated to contribute to the pool of free CKs within the organism. Based on phylogenetic analysis, *Dictyostelium* possesses three IPT genes: *iptA*, *iptB*, and *iptC*. *iptA* groups with adenylate-IPTs, and *iptB* and *iptC* are putative tRNA-IPTs [6,19,20]. Collectively, these three IPT genes appear to be distantly related to plant IPTs and were likely acquired through horizontal gene transfer events, in support of previous phylogenetic analyses [20,21]. The protein product of *iptA* facilitates the formation of iP-type CKs. The enzymes responsible for the synthesis of iP and DA (isopentenyltransferase, IptA; discadenine synthase) are considered to be developmentally regulated, as their peak activities have been identified following the onset of culmination (initiation of fruiting body formation) [6,22,23]. While CKs have a significant role in the later developmental stages of the *Dictyostelium* life cycle, much remains to be understood regarding the biosynthesis, signal transduction, and roles of CKs across the entire life cycle.

In this study, ultra high-performance liquid chromatography-positive electrospray ionization-high resolution tandem mass spectrometry (UHPLC-(ESI+)-HRMS/MS) was used to profile endogenous CKs during the 5 major stages of the *Dictyostelium* life cycle: growth, aggregation, mound, slug, fruiting body, and germination. Through comprehensive profiling of 30 naturally occurring CKs, this study reveals that *Dictyostelium* produces and secretes 6 CK forms in varying abundance throughout the life cycle, giving insight into CK biosynthesis among social amoebae. Additionally, the metabolome of each life cycle stage was investigated to identify any significant trends in CK metabolites in comparison to other small molecule metabolites secreted during development. Finally, the effect of CKs on *Dictyostelium* growth was determined through exogenous hormone application of three different CK types over a 144-h growth period. Collectively, these results provide evidence of an expanded role for CKs beyond the previously described functions limited to spore formation and dormancy.

## 2. Materials and Methods

### 2.1. Cell Lines, Buffers, and Chemicals

The *Dictyostelium* AX3 strain was obtained from the Dicty Stock Center, and it was initially grown and maintained at room temperature on SM agar with Klebsiella aerogenes [24]. Subsequent liquid cultures were grown axenically in either HL5 medium or FM minimal medium (Formedium, Hunstanton, Norfolk, UK) supplemented with ampicillin (100 µg/mL) and streptomycin sulfate (300 µg/mL) at 150 rpm and room temperature. All cells used in experiments were harvested in the mid-log phase of growth (1–5 × 10^6^ cells/mL). FM minimal medium was used to avoid high background CK levels originating from biological ingredients present in standard nutrient-rich medium (e.g., HL5). KK2 buffer (2.2 g/L KH_2_PO_4_ and 0.7 g/L K_2_HPO_4_, pH 6.5) was used as a starvation buffer and for washing cells harvested from petri dishes and agar plates. iP and *N*^6^-benzyladenine (BA) were purchased from Sigma-Aldrich (Oakville, Ontario, Canada) and were dissolved in a minimal volume of 1M NaOH and diluted with HPLC-grade methanol (CH_3_OH) to prepare a stock solution (0.1 mM) for exogenous treatment. DA was synthesized as described by Mik et al. [25] with slight modifications and was dissolved in 100% DMSO to prepare a stock solution (0.657 mM). The concentrations of methanol and DMSO in the growth medium did not exceed 1%.

### 2.2. Sampling of Life Cycle Stages for CK Profiling

Five life cycle stages representative for the key morphological changes exhibited throughout *Dictyostelium* growth and development were chosen for CK analysis: growth (single-celled amoeba), aggregation (6-h starvation in KK2 buffer), mound, slug, and fruiting body. For all life cycle stages, KK2 buffer was separated from the cell pellet by centrifugation to allow for analysis of intracellular CK retention (IC) and extracellular secretion (EC).

For the growth and aggregation samples, 6.25 × 10^6^ growth-phase cells were plated in polystyrene Petri dishes (100 mm × 15 mm) and grown overnight at room temperature in 7 mL of FM minimal medium. The following day, the medium from the growth samples was harvested separately from the adherent cells in the Petri dish. The EC samples were centrifuged for 5 min at 986g to remove cell debris, transferred to a new sterile 15 mL tube, and stored immediately at −20 °C until CK metabolites were extracted for UHPLC-(ESI+)-HRMS/MS analysis. The remaining adherent cells were removed from the Petri dish by pipetting, washed twice with KK2 buffer, pelleted for 1 min at 6,200g, and stored immediately at −20 °C. Cell concentration was determined for each replicate using a hemocytometer. For the aggregation samples, the FM minimal medium was discarded and replaced with 7 mL of KK2 buffer for 6 h. After 6 h, the KK2 buffer was collected as the EC samples, and the cells were pelleted, as described for the growth samples to analyse the IC CK content.

For the developmental life cycle stages (mound, slug, and fruiting body), 5 × 10^7^ growth-phase cells suspended in 300 µL of KK2 buffer were spread evenly onto 1% water agar and maintained in the dark in a humidity chamber for 10–24 h. The individual plates were harvested only if there was synchronous development of the respective life cycle stage, where at least 80% of the developmental structures were alike. In general, mounds were observed after 10 h of development, slugs after 16–18 h, and fruiting bodies after 24 h. For all life cycle stages cultured on water agar, the respective structures were harvested by gently scraping the agar surface and washing it with 3 mL of KK2 buffer. The collected cells and KK2 buffer were centrifuged for 5 min at 986g. The KK2 buffer was carefully transferred to a new sterile 15 mL tube without disturbing the cell pellet and then stored separately at −20 °C to be analysed as the EC samples. The remaining pellet for each developmental life cycle stage was immediately stored at −20 °C to be analysed as the IC samples.

### 2.3. Spore Germination

Spore germination was assayed using FM minimal medium and 1% water agar. First, 7.5 × 10^7^ growth-phase cells suspended in 300 µL of KK2 were evenly spread onto water agar and allowed to form fruiting bodies as described above. The spores were harvested by collecting the entire fruiting body structures as follows: (1) the agar surface was washed with 1 mL KK2 buffer and gently scraped with a cell scraper, (2) the KK2 buffer containing the entire fruiting body structure and spores was placed into a sterile microcentrifuge tube, (3) the spore suspension was centrifuged for 5 min at 986g to pellet the samples (a sample was collected prior to centrifugation to determine cell concentration using a hemocytometer), (4) the buoyant cellulose stalks were removed with a pipette tip, and (5) the remaining spores were plated in 7 mL of FM minimal medium. The spores took roughly 72 h to germinate (> 75%). During this timeframe, IC and EC samples were collected separately at three different time points: 24-, 48-, and 72-h. The 7 mL of medium were separated from the spores and/or germinated amoebae at each of selected time points and were centrifuged for 5 min at 986g. Following centrifugation, the cell-free medium was transferred into separate 15 mL tubes to avoid any contamination from cellular debris and was stored at −20 °C to be processed for CK extraction as the EC samples. The remaining spores or amoebae were harvested from the Petri dish using a pipette, washed twice with KK2 buffer, re-pelleted, and then stored at −20 °C to be processed as the IC samples. Images were taken at each of the sampled life cycle stages to determine percent germination for each replicate.

### 2.4. Extraction and Purification of Dictyostelium-Derived CKs

A modified protocol of CK hormone extraction and purification optimized to minimize enzymatic activities causing CK nucleotide degradation and CK isomerization was used as described previously [26,27,28]. One mL of ice-cold (−20 °C) modified extraction solvent Bieleski No. 2 (CH_3_OH:H_2_O:HCO_2_H (15:4:1, *v*/*v*/*v*)) was added to the EC and IC samples. All samples were consecutively spiked with 10 ng of each of the deuterated internal standards to enable endogenous hormone quantification through isotope dilution. The following standards were added to each sample: ^2^H_6_[9RMP]DZ, ^2^H_6_[9RMP]iP, ^2^H_5_[9R]*t*Z, ^2^H_3_[9R]DZ, ^2^H_6_[9R]iP, ^2^H_7_[9R]BA, ^2^H_3_DZ, ^2^H_6_iP, ^2^H_7_BA, ^2^H_5_*t*ZOG, ^2^H_7_DZOG, ^2^H_5_*t*ZROG, ^2^H_7_DZROG, ^2^H_5_*t*Z7G, ^2^H_5_*t*Z9G, ^2^H_3_DZ9G, ^2^H_5_2MeS*t*Z, ^2^H_6_2MeSiP, ^2^H_5_2MeS*t*ZR, and ^2^H_6_2MeSiPR (Olchemim Ltd., Olomouc, Czech Republic, Table 1). Isotopically-labelled internal standards (IS) were not available for all analytes, so in some cases an IS was used for more than 1 analyte (Table 1). In the case of DA, (in which no commercial labelled standard was available), the presence of DA in sample extracts was confirmed by examining the isotopic fine structure in mass spectra obtained with a Bruker SolariX XR, Fourier transform ion cyclotron resonance mass spectrometer (FTICR-MS) (Billerica, MA, USA) equipped with a 7T magnet (Supplementary Materials, Figure S1). The samples were allowed to passively extract overnight at −20 °C in the modified Bieleski No. 2 extraction solvent. The following day, the samples were centrifuged (11,180g for 10 min), and the supernatant was collected and placed into a new tube. The remaining IC and EC samples were resuspended in an additional 1 mL of the extraction solvent, and the samples were allowed to extract for an additional 30 min at −20 °C. Re-extracted samples were centrifuged and both supernatants were pooled and evaporated to in a speed vacuum concentrator (Savant SPD111V, UVS400, Thermo Fisher Scientific, Waltham, MA, USA) at room temperature.

Dried extraction residues from both the EC and IC samples were reconstituted in 1 mL of 1 M HCO_2_H (pH 1.4) to ensure complete protonation of all CKs. Each extract was subjected to solid phase extraction (SPE) on a mixed mode, reversed-phase, cation-exchange cartridge (MCX 6cc; 200 mg, Canadian Life Sciences, Peterborough, ON, Canada). Cartridges were activated with 5 mL of CH_3_OH and equilibrated with 5 mL of 1M HCO_2_H. Each sample was loaded and allowed to pass through the column by gravity and the columns were then washed with 5 mL of 1 M HCO_2_H, followed by 5 mL of CH_3_OH. The nucleotide fraction was eluted first using 5 mL of 0.35 M NH_4_OH to be processed separately from the other CK fractions, which are retained on the column based on charge and their hydrophobic properties. Free bases, ribosides, methylthiols, and glucosides were subsequently eluted together using 5 mL of 0.35 M NH_4_OH in 60% CH_3_OH. Both eluted fractions were evaporated in a speed vacuum concentrator at room temperature and stored at −20 °C until further processing.

Since CK-nucleotides could not be directly analysed by our LC-MS/MS method, the separately eluted nucleotide samples were dephosphorylated using 3 units of alkaline phosphatase (alkaline phosphatase calf intestine, New England BioLabs, Whitby, ON, Canada) in 1 mL of 0.1 M ethanolamine-HCl (pH 10.4) for 12 h at 37 °C [29]. The resulting ribosides were evaporated in a speed vacuum concentrator at room temperature. The riboside fraction obtained as a result of this processing reflects the pooled contribution of mono-, di-, and tri-phosphates of each CK type (iPRP, *c*ZRP, *t*ZRP, and DZRP). Samples were reconstituted in 1.5 mL Milli-Q H_2_O for further purification on C_18_ cartridges (C_18_ 6cc, 500 mg, Canadian Life Sciences; Peterborough, ON, Canada). Cartridges were activated with 3 mL of CH_3_OH and equilibrated with 6 mL of Milli-Q H_2_O. Samples were loaded onto the C_18_ cartridge and were allowed to pass through the column by gravity. The residue was washed with 3 mL of Milli-Q H_2_O and the ribosides were eluted using 1.25 mL of CH_3_OH:H_2_O (80:20 *v*/*v*). All sample eluents were evaporated, and residues were stored at −20 °C until further processing.

Prior to UHPLC-(ESI+)-MS/MS analysis, the extracted CK fractions were re-constituted in 1.5 mL of initial mobile phase conditions (95:5 H_2_O:CH_3_CN) both with 0.08% CH_3_CO_2_H. Samples were transferred to glass auto-sampler vials and stored at −20 °C until analysis.

### 2.5. CK Quantification by UHPLC-(ESI+)-HRMS/MS

*Dictyostelium* CKs within the EC and IC samples were identified and quantified using UHPLC-(ESI+)-HRMS/MS [30]. Briefly, a 25 µl sample volume was injected into the Thermo Ultimate 3000 UHPLC coupled to a Thermo Q-Exactive^™^ Orbitrap mass spectrometer equipped with a heated electrospray ionization (HESI) source (Thermo Scientific, San Jose, CA, USA). Compounds were separated using a reversed-phase C_18_ column (Kinetex 2.6 µ C18 100 A, 2.1 × 50 mm; Phenomenex, Torrance, CA, USA). All hormone fractions were eluted with a multistep gradient of component A: H_2_O with 0.08% CH_3_CO_2_H mixed with component B: CH_3_CN with 0.08% CH_3_CO_2_H at a flow rate of 0.4 mL/min. The initial conditions were 5% B increasing linearly to 10% B over 2 min followed by an increase to 95% B over 6.5 min; 95% B was held constant for 1.5 min before returning to starting conditions for 5 min; total run time was 15 min.

The eluate was introduced into the Orbitrap HESI source (capillary temperature of 250°C) and analysed using parallel reaction monitoring (PRM) at 35,000 resolution. CKs were analysed in positive ion mode. The HESI source was operated with sheath gas, 30 arbitrary units; auxiliary gas, 8 arbitrary units; max spray current, 100 µA; auxiliary gas heater temperature, 450 °C; S-lens RF level, 60 and spray voltage 3.9 kV. The PRM parameters included the following: automatic gain control (AGC), 1 × 10^6^; maximum injection time (IT), 128 ms; *m*/*z* 1.2 isolation window and normalized collision energy (NCE) individually optimized for each CK analyte.

All data was analysed using Thermo Xcalibur (v 3.0.63) software (Thermo Scientific, San Jose, CA, USA), to calculate peak areas. Quantification was achieved through isotope dilution analysis based on recovery of ^2^H-labelled internal standards.

### 2.6. Untargeted Metabolomics 

The samples extracted for CK profiling from the developmental stages were further used for untargeted metabolomics analysis. The extraction protocol used MCX cartridges, which are specific for basic, polar compounds, such as CKs. The metabolomic data were acquired in full scan (FS) mode using the Thermo Q-Exactive^™^ Orbitrap mass spectrometer. The samples were injected into the mass spectrometer through the Thermo Ultimate 3000 UHPLC system using the parameters described in Section 2.5. For FS analysis, each sample was run in positive ion mode over the mass range of *m*/*z* 80−600, at 35,000 resolution, with automatic gain control (AGC) target of 3 × 10^6^, and maximum injection time (IT) of 128 ms.

The raw data files were uploaded directly to XCMS Online [31,32] to analyse significantly up- or down-regulated *Dictyostelium* metabolites (including CKs) across the developmental stages (aggregation, mound, slug, and fruiting body) using the previously described settings [33] with slight modifications. Metabolite detection was achieved through the centWave method with a tolerance of 2.0 ppm [31]. KK2 buffer was used as a negative control to eliminate the metabolites present in the wash buffer from statistical analysis. The pre-filter was set to 6 scans with a minimum of 5000 intensity, the signal to noise threshold was 10, and noise was set to 1 × 10^6^ for positive mode. The obiwarp method was used for retention time correction [34]. The ‘fillPeaks’ function with default settings and remaining zeros were imputed with two-thirds the minimum value on a per mass basis. Compounds produced by *Dictyostelium* were identified by accurate mass using the METLIN database.

### 2.7. Growth Assays with Exogenous CK Treatment

Cells in the mid-log phase of growth (1–5 × 10^6^ cells/mL) were collected and resuspended to a final concentration of 1 × 10^6^ cells/mL in 5 mL of fresh HL5 medium. The cells were incubated at room temperature and 150 rpm over a 6-day period. Cell concentrations were assessed daily using a hemocytometer. HL5 medium was supplemented with iP, BA, or DA to determine the effect of CKs on cell proliferation at the following concentrations: 0 nM, 1 nM, 10 nM, 100 nM, 500 nM, and 1 µM. Statistical significance was assessed in GraphPad Prism (GraphPad Software Incorporated, La Jolla, CA, USA) using two-way ANOVA followed by Bonferroni’s multiple comparisons test (*p* values < 0.05 were considered significant; *n* = the number of biological replicates analysed).

## 3. Results

### 3.1. CKs Are Detected Throughout All Stages of the Dictyostelium Life Cycle

To determine the ability of *Dictyostelium* to synthesize diverse CK forms during its life cycle, a total of 30 naturally occurring CKs were profiled by UHPLC-(ESI+)-HRMS/MS during the 5 stages of the *Dictyostelium* life cycle: growth (single-celled amoeba), aggregation (induced by 6-h starvation), mound, slug, and fruiting body. An analysis of *Dictyostelium* culture media was conducted prior to the CK profiling experiments to determine which medium contained the lowest background levels of CKs (Supplementary Materials, Figure S2). From this analysis, FM minimal medium was selected since it contains the lowest levels of background CK levels compared to other more traditional nutrient rich media, such as HL5.

**Figure 1:**
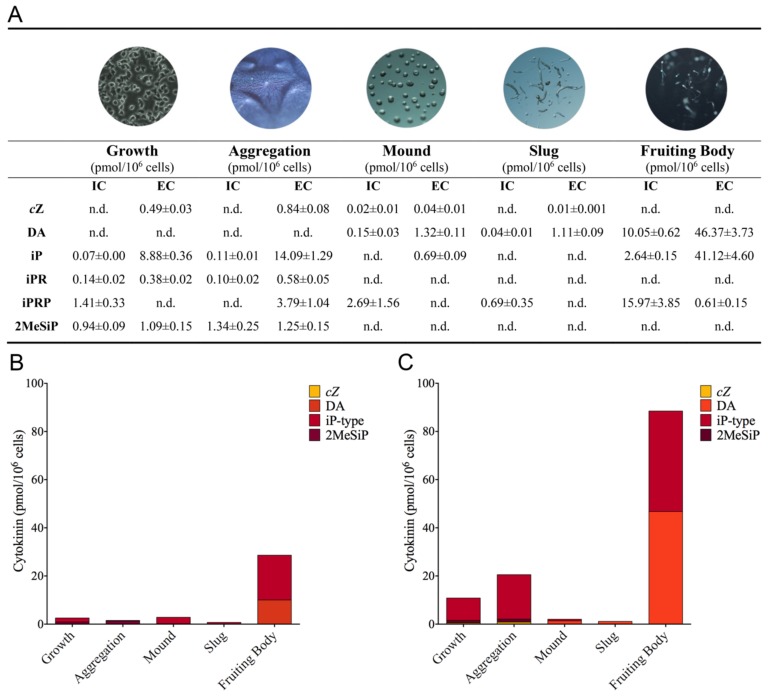
Cytokinin (CK) production (pmol/10^6^ cells) detected by ultra high-performance liquid chromatography-positive electrospray ionization-high resolution tandem mass spectrometry (UHPLC-(ESI+)-HRMS/MS) during 5 stages of the *Dictyostelium discoideum* life cycle. (**A**) Individual CK analyte concentrations detected intracellularly (IC) and extracellularly (EC) during the life cycle. Values presented as means ± standard error of the mean (SEM; *n* = 4); n.d. represents CKs not detected. (**B**) Total concentrations of CK-types detected within the IC samples at the 5 life cycle stages. (**C**) Total concentrations of CK-types detected within the EC samples. iP-type CKs encompass the free base (iP), riboside (iPR), and nucleotide (iPRP).

Our CK profiling analysis revealed that *Dictyostelium* synthesizes 6 CK forms in varying concentrations throughout the different life cycle stages: cZ, DA, iP, *N*^6^-isopentenyladenosine (iPR), *N*^6^-isopentenyladenine-9-riboside-5′ phosphate (iPRP), and 2-methylthio-*N*^6^-isopentenyladenine (2MeSiP) (Figure 1A). Among the profiled stages, the fruiting body contained the highest levels of CKs (total IC CK: 28.66 pmol/10^6^ cells; total EC CK: 88.47 pmol/10^6^ cells). Here, in addition to detecting iP and DA, we identified the nucleotide form of iP, iPRP, in both the IC and EC samples at concentrations of 15.97 pmol/10^6^ cells and 0.61 pmol/10^6^ cells, respectively (Figure 1A).

During growth and aggregation, the CK profiles were dominated by iP-type CKs: iP, iPR, and iPRP (Figure 1B,C). Among the CKs detected in both stages, iP free base was synthesized and detected at the highest concentration in the EC samples. Interestingly, the concentration of iP increased most dramatically from growth (8.88 pmol/10^6^ cells) to aggregation (14.09 pmol/10^6^ cells) in the EC samples (Figure 1A). iPR was present at low concentrations (< 0.60 pmol/10^6^ cells) both intracellularly and extracellularly, with a general trend of the EC concentration being 3 and 6 times higher than that of the IC concentration in the growth and aggregation stages, respectively (Figure 1A). iPRP was only detected in the IC samples during growth and in the EC samples during aggregation. *c*Z and 2MeSiP were also detected in both of these stages. *c*Z was consistently detected in the EC samples in low concentrations (< 0.90 pmol/10^6^ cells), and 2MeSiP was detected at similar IC and EC concentrations (between 0.94 and 1.35 pmol/10^6^ cells). DA was not detected during growth or aggregation.

CKs were detected in the least abundance during the mound and slug life cycle stages (Figure 1). The total level of CKs did not exceed a concentration of 3 pmol/10^6^ cells for either the IC or EC samples. *c*Z, DA, and iPRP were present in both the mound and slug stages, whereas iP was only detected in the EC samples during mound formation. The EC values for these stages may be slightly underestimated compared with the other life cycle stages, as these structures were harvested directly off of the agar surface, and the wash buffer was collected as the EC samples.

### 3.2. DA Is One of the Most Up-Regulated Metabolites during Dictyostelium Development

Following our CK profiling results, we examined the metabolome (all detectable small molecules restricted to our sample preparation conditions) of the *Dictyostelium* life cycle. This enabled further identification of trends in CK analytes in comparison to other detected non-CK metabolites using an untargeted, high-resolution Orbitrap LC-MS analysis—followed by XCMS feature detection and multigroup analysis through XCMS Online [32]. This dataset has been made available through XCMS Online (ID: 1201031). Within the EC samples collected at the aggregation, mound, slug, and fruiting body stages, a total of 476 common metabolite features were detected. Of these 476 aligned metabolite features, 276 were significantly different among the sampled developmental time points (> 1.5-fold change, *p* < 0.01). DA was among the most upregulated metabolites during *Dictyostelium* development (Supplementary materials, Figure S3). Specifically, DA accumulated most dramatically from the slug to fruiting body life cycle stage and was the 31st most significantly changed feature during development (*p* = 3.73 E^−12^). Beyond DA, there were no other CK analytes identified as a feature significantly up- or down-regulated relative to the other small molecule metabolites identified among the analysed *Dictyostelium* life cycle stages (data not shown).

### 3.3. CK Levels Are Highest During Germination

**Figure 2:**
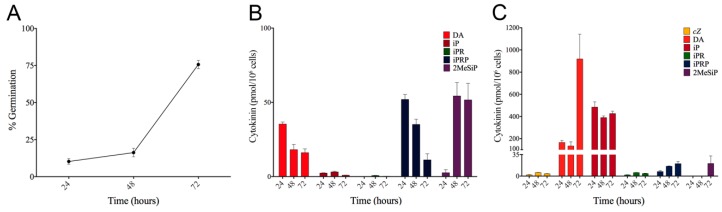
*Dictyostelium* germination rate (%) and cytokinin (CK) production over a 72-h time course. (**A**) The percentage of germinated spores was determined for each of the sampled time points. (**B**) IC CK analyte concentrations detected by ultra high-performance liquid chromatography-positive electrospray ionization-high resolution tandem mass spectrometry (UHPLC-(ESI+)-HRMS/MS) during a 72-h germination period. (**C**) EC CK analyte concentrations detected by UHPLC-(ESI+)-HRMS/MS during a 72-h germination period. The presented values are means ± SEM (*n* = 4). The experiment shown was a typical response that was confirmed by other independently replicated trials.

Considering the role of CKs to maintain spore dormancy paired with our observation of high CK levels during the fruiting body life cycle stage, we assessed CK synthesis during the process of spore germination over a 72-h time course. Percent germination was determined over the 72-h time course, in which > 75% of the spores germinated by the final 72-h sampling period (Figure 2A). After 24-, 48-, and 72-h in FM minimal medium, IC and EC samples were harvested to extract CKs. Five CK forms were detected in the IC germination samples (DA, iP, iPR, iPRP, and 2MeSiP; Figure 2B). In the EC samples, the same five CK forms were detected plus an additional CK form, *c*Z (Figure 2C).

During all sampled life cycle stages, *Dictyostelium* secreted more CKs outside of the cell (EC samples) than it retained within the cell (IC samples) (Figure 1 and Figure 2). During germination, the concentrations of DA and iP were the highest of the 6 detected CK analytes (Figure 2). The IC samples were largely dominated by iPRP at the 24-h sampling period, which slowly decreased by nearly 5-fold over the germination time course (Figure 2B). Inversely, 2MeSiP production increased dramatically over the germination period. DA production within the IC samples was highest when approximately 10% of the spores had germinated and slowly decreased as germination progressed. Lastly, IC levels for iP and iPR were relatively low and unchanging (< 3 pmol/10^6^ cells) over the 72-h time course (Figure 2B).

The EC germination samples contained the highest concentrations of CK in all the sampled life cycle stages (Figure 2C). Specifically, iP and DA were the most abundant CK analytes, with concentrations 10- and 19-times higher, respectively, than those observed in the fruiting body life cycle stage. These 2 CKs had differing trends over the 72-h time course; DA increased in concentration from 166 pmol/10^6^ cells to 919 pmol/10^6^ cells, whereas, iP concentrations decreased slightly from 485 pmol/10^6^ cells at the 24-h time point to 426 pmol/10^6^ cells at the 72-h time point (Figure 2C). Similar to DA, iPRP concentration also had an increasing trend over the 72-h germination period, where the initial concentration of 7 pmol/10^6^ cells nearly tripled to 20 pmol/10^6^ cells. 2MeSiP was only detected at the final 72-h time point, when over 75% of the spores had germinated. Lastly, *c*Z and iPR concentrations were detected at low, unchanging levels (< 6 pmol/10^6^ cells; Figure 2C).

### 3.4. N^6^-Isopentenyladenine Prolongs the Stationary Phase of Dictyostelium Growth

**Figure 3:**
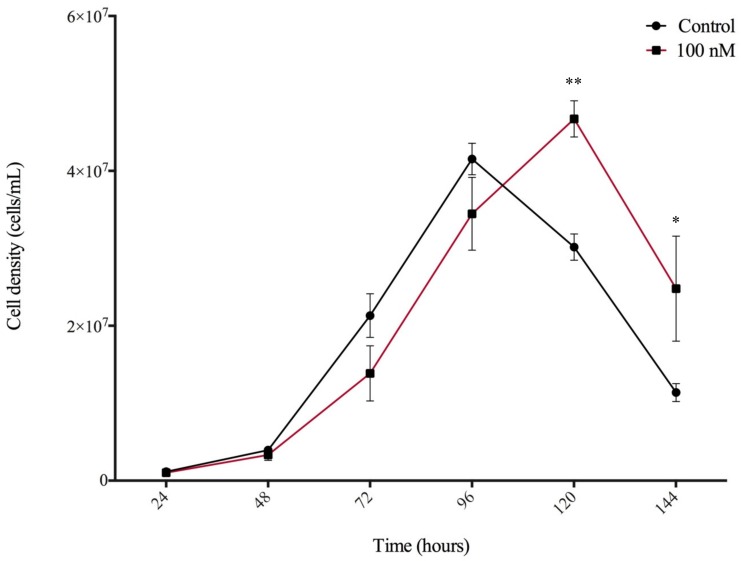
Effect of 100 nM *N*^6^-isopentenyladenine (iP) treatment on AX3 cell proliferation in HL5 medium over a 144-h growth period. Data are presented as mean concentration (cells/mL) ± SEM (*n* = 6). Statistical significance was assessed using two-way ANOVA (*p* < 0.05) followed by the Bonferroni multiple comparisons test. This analysis revealed a significant effect of CK treatment on growth for the 100 nM concentration of iP at the 120-h and 144-h time points (** *p* < 0.01, and * *p* < 0.05). This finding was replicated in two independent experiments.

Considering the detection of CKs during *Dictyostelium* growth, the effect of exogenously applied CKs on the rate of cell proliferation in axenic culture was assessed. HL5 medium was supplemented with iP, DA, or BA at 5 different concentrations: 1 nM, 10 nM, 100 nM, 500 nM, and 1 µM. In order to test the CK effect at physiologically relevant concentrations, we did not exceed concentrations over 1 µM. Proliferation of the CK-treated AX3 cells was assessed over 6 days and compared against the proliferation of untreated cells. Interestingly, the CK analyte detected endogenously at the highest concentration during growth, iP, was the only CK to have an effect on cell proliferation, when supplemented to the medium. A significant difference in cell density was observed for the 100 nM iP-treated cells at the 120- and 144-h time points compared to untreated cells (*p* < 0.05) (Figure 3). No significant differences in cell proliferation were observed for cells treated with DA or BA at any of the 5 concentrations tested (data not shown).

## 4. Discussion

**Figure 4:**
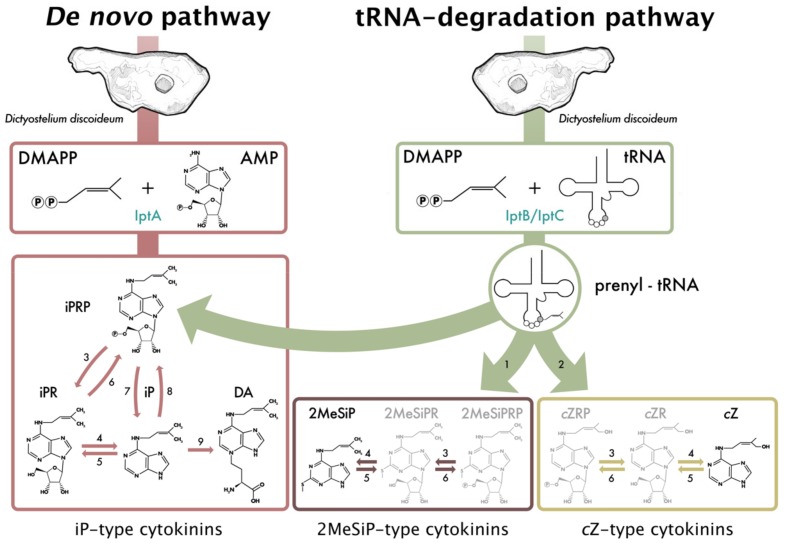
Proposed model of cytokinin (CK) biosynthesis in *Dictyostelium discoideum* consisting of two activation pathways—*de novo* and tRNA degradation. The information presented in the pathway was inferred from the current study and previous *Dictyostelium*, plant, and fungi studies [6,13,14,18,20,35,36,37]. Isopentenyltransferase (IptA) catalyses the addition of a prenyl group from dimethylallyl pyrophosphate (DMAPP) to adenosine monophosphate (AMP) to form free N^6^-isopentenyladenine-type (iP-type) CKs and discadenine (DA) via the de novo biosynthesis pathway [14,37]. IptB and IptC are proposed tRNA isopentenyltransferases which catalyze prenylation of tRNA molecules that can be further modified to form 2-methylthio-*N*^6^-isopentenyladenine-type (2MeSiP-type) CKs or *cis*-zeatin-type (*c*Z-type) CKs via the tRNA degradation pathway [6,20]. Degradation from the tRNA molecule contributes to the pool of free CKs, depicted by the three green arrows coming off of the prenyl-tRNA molecule. Expression data for the three isopentenyltransferase genes can be found in the Supplementary Materials (Figure S4) [38]. Black CK molecules depict the 6 CKs synthesized by *Dictyostelium* in the present study, while gray CK molecules represent CKs typical of CK biosynthesis pathways, but not detected in this study. Numbers represent inferred enzymes as follows: 1. cdk5rap1-like ortholog (DDB_G0287079); 2. *cis*-hydroxylase; 3. 5′-ribonucleotide phosphohydrolase; 4. adenosine nucleosidase; 5. purine nucleoside phosphorylase; 6. adenosine kinase; 7. CK phosphoribohydrolase (LOG-like ortholog, DDB_G0281309); 8. adenine phosphoribosyltransferase; 9. discadenine synthase.

This study provides insight into the nature of CK biosynthesis, secretion, and function during *Dictyostelium* growth, development, and spore germination. While the presence of CKs in *Dictyostelium* has been known for almost 40 years, CK detection within *Dictyostelium* was limited to only the fruiting body life cycle stage [7]. Considering the dominant presence of iP and DA within *Dictyostelium* and that iP CKs are the precursor molecules from which other CK types can be formed, UHPLC-(ESI+)-HRMS/MS was used in this study to determine whether additional CK forms are present in *Dictyostelium* throughout the other, not previously investigated life cycle stages. This study reveals that CK metabolites are synthesized by *Dictyostelium* during growth and development, beyond what has been previously documented. Specifically, the CK analytes *c*Z, DA, iP, iPR, iPRP, and 2MeSiP were detected. To our knowledge, endogenous production of *c*Z, iPR, iPRP, and 2MeSiP has never been documented in *Dictyostelium*, and we are the first to document the production of CKs outside of the fruiting body life cycle stage. These CK profiling results expand our understanding of CK metabolism in *Dictyostelium* and allow us to propose a model of CK biosynthesis (Figure 4).

Of the iP-type CKs identified in this study, the free base, iP, was the most abundant CK analyte, with the exception of the mound stage. The presence of the nucleotide, riboside, and free base iP forms suggests that *Dictyostelium* follows a similar isoprenoid CK biosynthesis pathway, as found in plants and other CK-producing organisms (Figure 4) [13,18,35]. An early report on the cell free CK biosynthesis in *Dictyostelium* was conducted by Taya et al. [37] who utilized crude extracts harvested from the late stages of *Dictyostelium* culmination to determine that 5′AMP was the acceptor molecule of the isopentenyl group onto the *N*^6^ position of the adenine. Radio-labelled AMP and unlabelled isopentenyl pyrophosphate (IPP) were assayed with crude *Dictyostelium* extracts, in which iPRP, iPR, and iP were formed as products and were identified through thin-layer chromatography. iPRP was shown to be the first reaction product; however, the authors concluded that further studies were necessary to determine whether iP is formed directly from iPRP or if iPR acts as an intermediate. While that early study demonstrated that *Dictyostelium* extracts possess the enzymes responsible for CK biosynthesis, it did not detect endogenous levels of iPRP or iPR. The detection of the nucleotide and riboside fractions of iP in the present study, in all life cycle stages where iP is detected, suggests that iP-biosynthesis in *Dictyostelium* utilizes iPR as an intermediate form between the nucleotide and free base iP forms, as shown in our proposed biosynthesis figure (Figure 4).

It is established that iP acts as the precursor molecule from which DA is synthesized [14]. The presence of the iP nucleotide, riboside, and free base forms during growth and aggregation, even when no presence of DA was detected, suggests iP-type CK have additional biological functions beyond acting as the substrate for DA production. In support of this finding, iP was the only one of the exogenously applied CKs to stimulate the *in vitro* growth of AX3 cells. This coincides with our CK metabolite results, which showed the free base iP concentration considerably increased from growth to aggregation. We would expect to see an enhanced effect of exogenous iP treatment on *in vitro* growth in IPT knockout cell lines in the absence of endogenous CK production. Within CK-producing organisms, there are often many functions carried out by a single CK molecule; for instance, in fungi, endogenously produced CKs affect nutrient uptake and water and ion transport [39,40]. Within *Dictyostelium*, further investigations looking at interactions between iP, cAMP, and nitric oxide (NO) may determine if CKs play a role in regulating either of these important molecules that facilitate the transition to multicellularity.

*c*Z was detected during growth and early development at low levels in the EC and the IC samples (< 1 pmol/10^6^ cells). During germination, the level of *c*Z was slightly elevated compared to that detected during growth and development. Of the isoprenoid-type CKs, the role of *c*Z is the least understood owing to its lower activity in many classical CK bioassays compared to its *trans*-Zeatin (*t*Z) isomer [41]. Synthesis of *c*Z is believed to originate from the mevalonate pathway via tRNA-IPTs [42]. Specifically, tRNA-IPTs prenylate position A37 of a specific subset of tRNA molecules forming iP-bound tRNA. A hydroxylating enzyme further modifies the iP side chain to form *c*Z-bound tRNA molecules, which is depicted as number 2 in the proposed CK biosynthesis pathway (Figure 4). Considering that the *Dictyostelium* genome contains 2 putative tRNA-IPTs (*iptB* and *iptC*), it follows that the detected levels of *c*Z are likely a result of degradation of the tRNA molecules modified through tRNA-IPT and a hydroxylating enzyme. However, further research is necessary to confirm the source of *c*Z synthesis and whether these low levels are of biological significance to *Dictyostelium* growth and development.

Along with *c*Z, 2MeSiP was detected during growth, aggregation, and germination. The levels of 2MeSiP were lower during growth and aggregation (< 2 pmol/10^6^ cells) compared to germination (between 2–55 pmol/10^6^ cells). In comparison to other CK-types, the role of methylthio-CKs (2MeSCKs) is the most obscure. These CKs are found in a subset of tRNAs as secondary modifications to tRNA-bound iP in plants, fungi, bacteria, and mammals. Upon tRNA degradation, the 2MeSCKs contribute to the free CK pool within the organism [35,43,44,45]. Collectively, CK-tRNA modifications are strongly linked to translation fidelity, but the activities of free, tRNA-derived CKs have never been characterized [see reviews [46,47]. In bacteria, the product of the *miaB* gene is responsible for methylthiolation of tRNA-bound iP, whereas the product of the less common gene, *miaE*, first isolated from *Salmonella typhimurium* is responsible for hydroxylation of 2MeSiP to 2MeSZ [48]. In mammals, the gene responsible for 2MeSiP modifications in tRNA is known as *CDK5RAP1* [49]. Interestingly, the *Dictyostelium* genome encodes a putative homolog of *CDK5RAP1* (depicted as enzyme 2 in the CK biosynthesis pathway, Figure 4), but the gene itself remains to be characterized (DDB_G0287079; http://www.dictybase.org). Based on this information, it is possible that 2MeSiP is produced via a pathway that is similar to the pathways observed in other CK-producing organisms.

CK profiles and metabolomic data support previous literature on the presence of DA in the fruiting body life cycle stage. Low levels of DA were detected in the mound and slug stages at concentrations 38-fold lower than those detected in the fruiting body. Interestingly, the data obtained from the metabolomics study suggest that DA is one of the most upregulated small molecules during development, which speaks to the importance of its tight regulation leading to rapid encapsulation of spores and maintenance of spore dormancy [6,15]. Similar to the spore dormancy effects of DA, a hypersporulation phenotype is observed in the fungi, *Claviceps purpurea*, following knockout of the CK-specific hydroxylating enzyme, Cpp450, which forms *t*Z [50]. As such, the role of CKs in the regulation of sporulation extends beyond what is observed in the Amoebozoa phylum.

Lastly, the spore germination assay expands upon previous research illustrating the continued production of DA during the process of germination. The increasing extracellular concentrations of DA support previous studies regarding the role of DA in germination inhibition; accordingly, the spores should cease intracellular DA production in the presence of available nutrients and, thereby, allow for DA secretion into the surrounding environment to rid the inhibition signal and allow germination of amoeba. This is supported by the IC data for DA, as the highest DA levels were detected at the 24-h time point and production decreased as germination occurred. The extracellular DA levels increased consistently over 72-h with the final time-point containing the highest level of CKs detected at any life cycle stage. From this data, there are many questions to pursue regarding the expanded roles of CKs and respective signal transduction pathways in *Dictyostelium*. This becomes especially pertinent following the identification of novel CK forms during *Dictyostelium* growth and development in the present study.

## 5. Conclusions

Collectively, the present results are the first to identify CKs in *Dictyostelium* beyond the fruiting body life cycle stage. In addition to iP and DA, four more CK analytes were identified in this study (*c*Z, iPR, iPRP, and 2MeSiP) defining the pathway by which *Dictyostelium* can synthesize multiple CK types and explains the origins of the two previously documented CK forms. The increase in iP free base during the transition from growth to development suggests a potentially wider role of CKs in the transition to multicellularity. Indeed, our experiments show that iP is the only tested CK form capable of increasing cell proliferation in liquid culture. Furthermore, the detection of the high CK levels during germination with distinct trends in DA synthesis support previous literature regarding the role of DA as a spore germination inhibitor. While much remains to be explored in *Dictyostelium* regarding the role of CKs in the life cycle, this study provides unique evidence of the CK production and supports previous literature on the involvement of CKs in the metabolism of non-plant organisms. This study expands our understanding of CK-producing organisms, thus highlighting the utility of the social amoeba in the research on this group of growth regulating hormones. Knockout experiments of the three isopentenyltransferase genes (*iptA*, *iptB*, and *iptC*) are underway to better resolve the biological roles of CKs in *Dictyostelium*.

## Figures and Tables

**Table 1:** Endogenous and ^2^H-labelled cytokinins (CKs) scanned for by ultra high-performance liquid chromatography-positive electrospray ionization-high resolution tandem mass spectrometry (UHPLC-(ESI+)-HRMS/MS) in *Dictyostelium* intracellular (IC) and extracellular (EC) samples. Labelled internal standards obtained from OlChemim Ltd. (Olomouc, Czech Republic) were used to identify and quantify CKs.

Endogenous CK Fractions	^2^H-labelled Internal Standard
**Nucleotides (RP)**	
*trans*-zeatin riboside-5′-monophosphate (*t*ZRP)	^2^H_6_[9RMP]DZ
*cis*-zeatin riboside-5′-monophosphate (*c*ZRP)	^2^H_6_[9RMP]DZ
Dihydrozeatin riboside-5′-monophosphate (DZRP)	^2^H_6_[9RMP]DZ
*N*^6^-benzyladenosine-5′monophosphate (BARP)	^2^H_6_[9RMP]DZ
*N*^6^-isopentyladenosine-5′monophosphate (iPRP)	^2^H_6_[9RMP]iP
**Ribosides (R)**	
*trans*-zeatin riboside (*t*ZR)	^2^H_5_[9R]*t*Z
*cis*-zeatin riboside (*c*ZR)	^2^H_5_[9R]*t*Z
Dihydrozeatin riboside (DZR)	^2^H_3_[9R]DZ
*N*^6^-isopentyladenosine (iPR)	^2^H_6_[9R]iP
*N*^6^-benzyladenosine (BAR)	^2^H_7_[9R]BA
**Free bases (FB)**	
*trans*-zeatin (*t*Z)	^2^H_3_DZ
*cis*-zeatin (*c*Z)	^2^H_3_DZ
Discadenine (DA)	^2^H_3_DZ
Dihydrozeatin (DZ)	^2^H_3_DZ
*N*^6^-isopentyladenine (iP)	^2^H_6_iP
*N*^6^-benzyladenine (BA)	^2^H_7_BA
**Glucosides (GLUC)**	
*trans*-Zeatin-O-glucoside (*t*ZOG)	^2^H_5_*t*ZOG
*cis*-Zeatin-O-glucoside (*c*ZOG)	^2^H_5_*t*ZOG
Dihydrozeatin-O-glucoside (DZOG)	^2^H_7_DZOG
*trans*-Zeatin-O-glucoside riboside (*t*ZROG)	^2^H_5_*t*ZROG
*cis*-Zeatin-O-glucoside riboside (*c*ZROG)	^2^H_5_*t*ZROG
Dihydrozeatin-O-glucoside riboside (DZROG)	^2^H_7_DZROG
*trans*-Zeatin-7-glucoside (*t*Z7G)	^2^H_5_*t*Z7G
*trans*-Zeatin-9-glucoside (*t*Z9G)	^2^H_5_*t*Z9G
*cis*-Zeatin-9-glucoside (*c*Z9G)	^2^H_5_*t*Z9G
Dihydrozeatin-9-glucoside (DZ9G)	^2^H_3_DZ9G
**Methylthiols (2MeS)**	
2-Methylthio-zeatin (2MeSZ)	^2^H_5_2MeS*t*Z
2-Methylthio-zeatin riboside (2MeSZR)	^2^H_5_2MeS*t*ZR
2-Methylthio-*N*^6^-isopentenyladenine (2MeSiP)	^2^H_6_2MeSiP
2-Methylthio-*N*^6^-isopentenyladenosine (2MeSiPR)	^2^H_6_2MeSiPR

Note: Bolded headings describing the various forms of cytokinin molecules below each heading.

## Supplementary Materials

The following are available online at https://www.mdpi.com/2218-273X/9/11/702/s1, Figure S1: Observed mass spectrum of *Dictyostelium discoideum* sample extract, Figure S2. Cytokinin (CK) profiles of three common *Dictyostelium discoideum* culture media: HL5, Lo-Flo (LF), and FM minimal medium, Figure S3. Extracted ion chromatogram for DA showing the peak intensity for each of the developmental life cycle stages of *Dictyostelium discoideum* analysed using a multigroup analysis performed through XCMS Online [32]. KK2 buffer was used as a negative control in the multigroup analysis to exclude features present in the buffer during statistical analysis.
