# Supplementary material for: Cytokinin Detection during the Dictyostelium discoideum Life Cycle: Profiles Are Dynamic and Affect Cell Growth and Spore Germination
Source: Biomolecules. 2019 Nov 5;9(11):702. doi: 10.3390/biom9110702 (PMC6920973; doi:10.3390/biom9110702)
Supplement: Supplementary file 1 [file biomolecules-09-00702-s001.pdf]

Supplementary Materials: Fourier transform ion cyclotron resonance mass spectrometer (FTICR-MS) Analysis: The presence of DA in sample extracts was investigated using a 7 T Bruker SolariX XR, FTICR-MS equipped with an electrospray ionization source operated in positive ion mode (Billerica, MA, USA). Sodium trifluoroacetic acid (0.1 mg/mL in methanol) was used to externally calibrate the instrument. Samples were infused at a rate of 180 μL/h and source parameters included capillary voltage of 5500 V, nebulizer gas pressure 1 bar, drying gas flow rate of 4 L/min and drying temperature of 200 °C. Accumulation time was optimized for 1×10^9 ions in the ion cyclotron resonance (ICR) cell. Mass spectra were acquired from *m/z* 54 to 1100 for 100 scans; 16 M data points were collected per scan with a free ion decay (FID) of 4.1943 s. Spectra were acquired using Bruker ftms Control software (version 2.1.0) and analysed using Bruker Compass DataAnalysis software (version 5.0). As indicated in Figure S1, the mass spectra obtained from a sample extract is in good agreement with the theoretical mass spectrum of discadenine (DA, $C_{14}H_{20}N_6O_2$), mass accuracy 0.066 ppm. Examination of the isotopic fine structure of the M^{+1} isotope (insert) are also in agreement with the theoretical mass spectrum of DA. The peak observed at *m/z* 306.17001 was not identified and is likely another analyte in the sample extract.

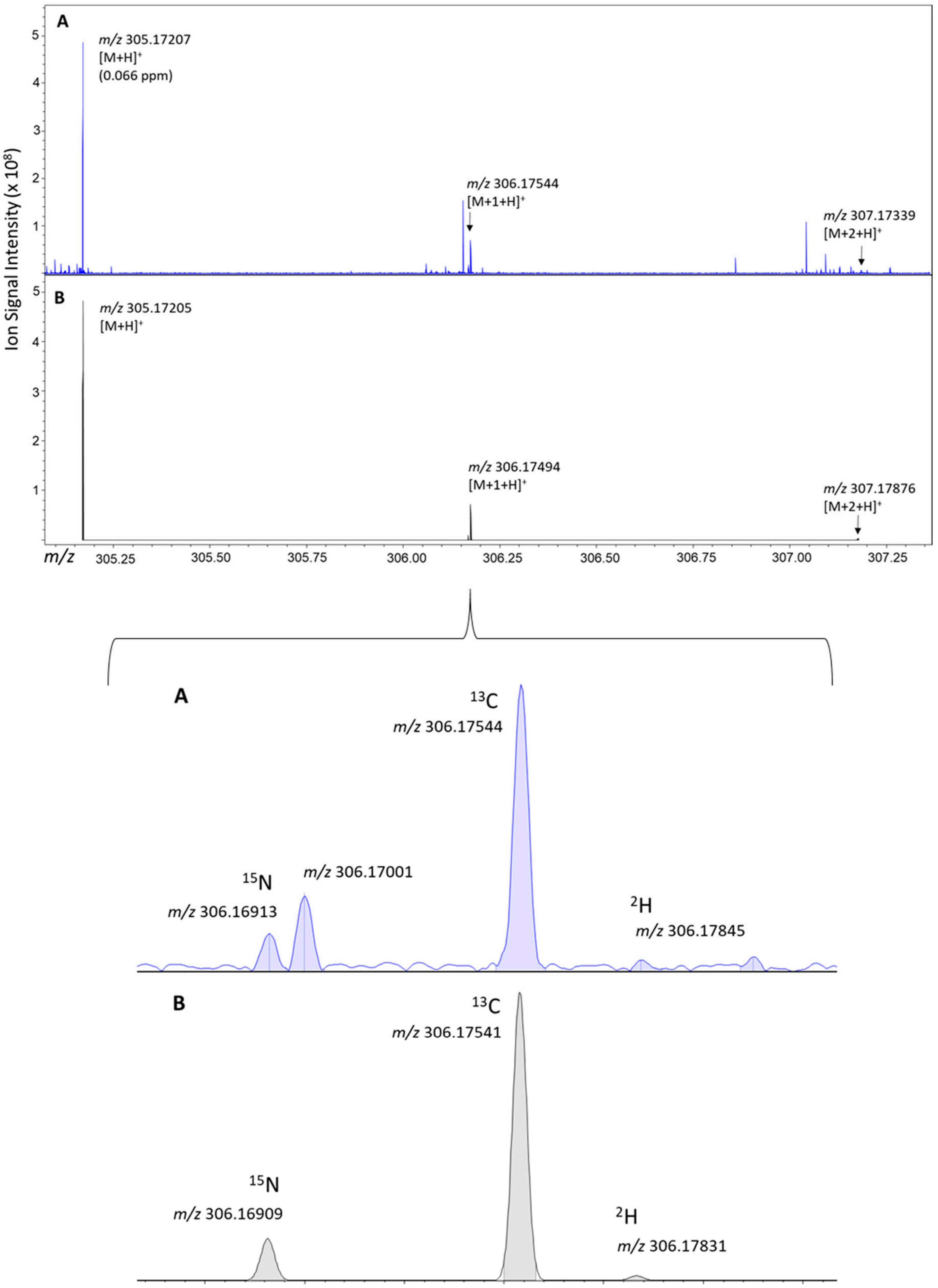

Figure S1. Observed mass spectrum of *Dictyostelium discoideum* sample extract (**A**) and the theoretical mass spectrum of discadenine (DA, $C_{14}H_{20}N_6O_2$) (**B**). Magnification of the isotopic fine structure of the M+1 isotope is shown.

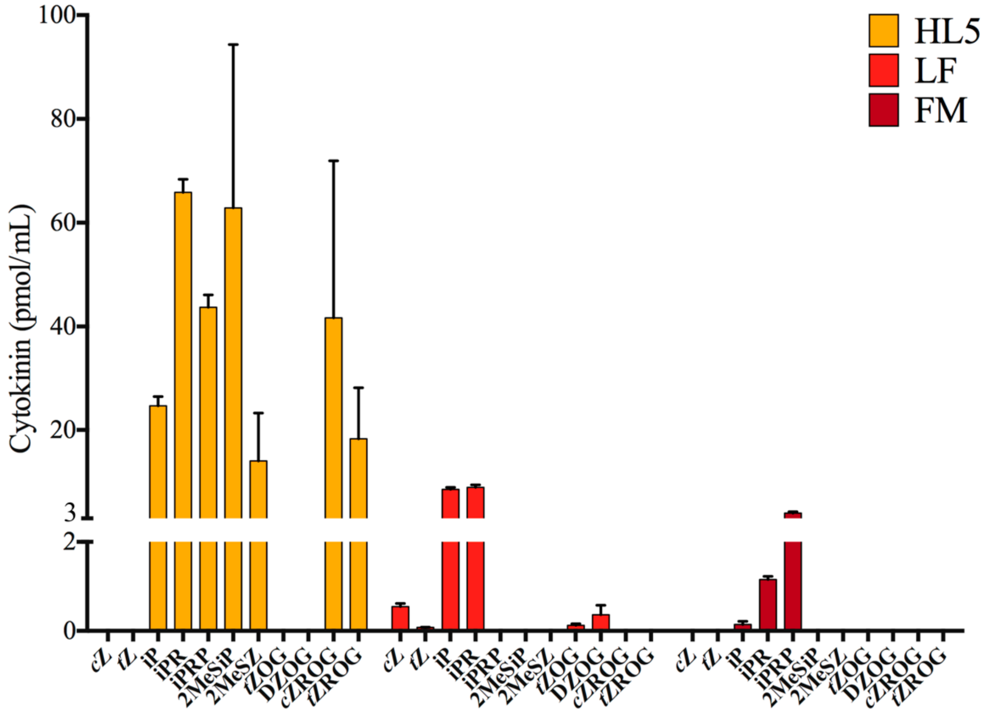

Figure S2. Cytokinin (CK) profiles of three common *Dictyostelium discoideum* culture media: HL5, Lo-Flo (LF), and FM minimal medium. Values presented as means ± standard error of the mean (SEM; *n*= 4).

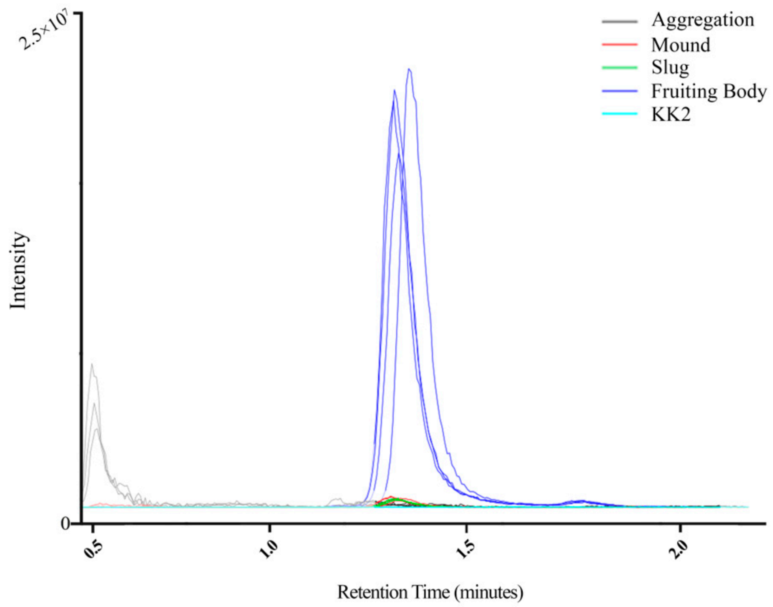

Figure S3. Extracted ion chromatogram for DA showing the peak intensity for each of the developmental life cycle stages of *Dictyostelium discoideum* analysed using a multigroup analysis performed through XCMS Online [32]. KK2 buffer was used as a negative control in the multigroup analysis to exclude features present in the buffer during statistical analysis. DA was eluted at 1.38 min at *m*/*z* 305.1705.

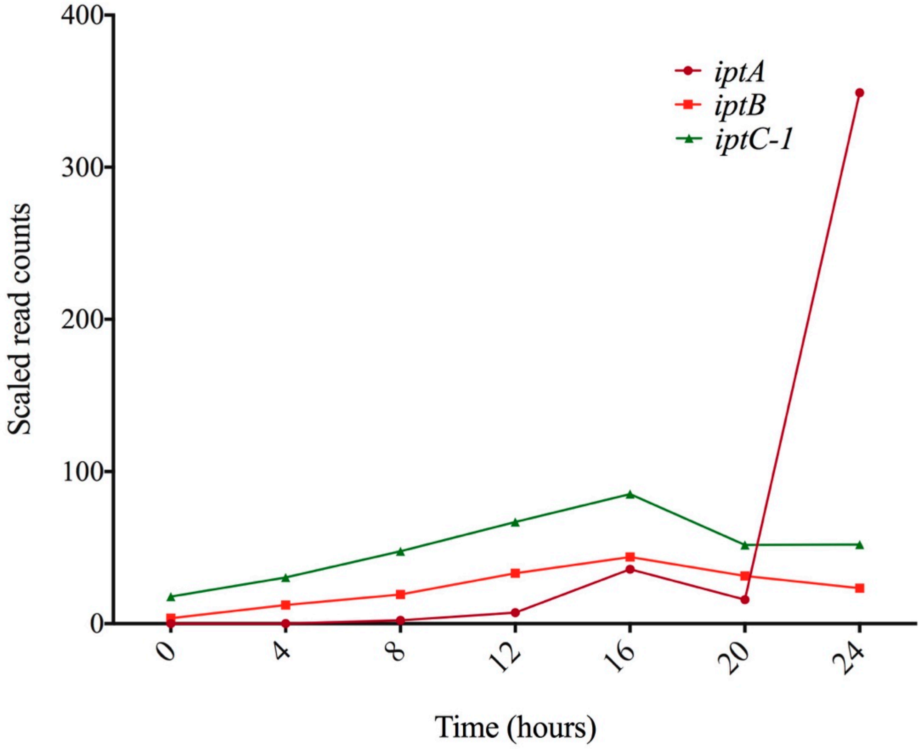

Figure S4. Gene expression analysis of the three isopentenyltransferase genes in *Dictyostelium discoideum*. RNA-Seq data was obtained from dictyExpress (www.dictyexpress.biolab.si) [38].
